# Direct Aminolysis of Ethoxycarbonylmethyl 1,4-Dihydropyridine-3-carboxylates

**DOI:** 10.3390/molecules201119697

**Published:** 2015-11-12

**Authors:** Brigita Vigante, Martins Rucins, Aiva Plotniece, Karlis Pajuste, Iveta Luntena, Brigita Cekavicus, Egils Bisenieks, Rufus Smits, Gunars Duburs, Arkadij Sobolev

**Affiliations:** Latvian Institute of Organic Synthesis, Aizkraukles str. 21, Riga LV-1006, Latvia; rucins@osi.lv (M.R.); aiva@osi.lv (A.P.); kpajuste@osi.lv (K.P.); iveta.vesere@gmail.com (I.L.); brigita.cekavicus@inbox.lv (B.C.); egils.bisenieks@osi.lv (E.B.); rufs@osi.lv (R.S.); gduburs@osi.lv (G.D.); and arkady@osi.lv (A.S.)

**Keywords:** 1,4-dihydropyridines, ethoxycarbonylmethyl esters, aminolysis, carbamoylmethyl esters, secondary amines, cyclic amines, 1,5,7-triazabicyclo[4.4.0]dec-5-ene

## Abstract

The ethoxycarbonylmethyl esters of 1,4-dihydropyridines were directly converted into carbamoylmethyl esters in the presence of 1,5,7-triazabicyclo[4.4.0]dec-5-ene (TBD) in good to excellent yields under mild conditions. The use of TBD is crucial for the successful aminolysis of ethoxycarbonylmethyl ester of 1,4-dihydropyridines with secondary amines as without it the reaction does not proceed at all. The aminolysis reaction proceeded regioselectively, as the alkyl ester conjugated with the 1,4-dihydropyridine cycle was not involved in the reaction. Screening of other *N*-containing bases, such as triethylamine (TEA), pyridine, 4-(*N*,*N*-dimethylamino)pyridine (DMAP), 1,8-diazabicyclo[5.4.0]undec-7-ene (DBU), 1,5-diazabicyclo[4.3.0]non-5-ene (DBN), imidazole, tetramethyl guanidine (TMG) and 7-methyl-1,5,7-triazabicyclo[4.4.0]dec-5-ene (MTBD) as catalysts revealed no activity in the studied reaction.

## 1. Introduction

As a privileged structure 1,4-dihydropyridine (1,4-DHP) represents an important scaffold for the design and development of novel pharmaceuticals [1]. Bis(alkyl) 4-aryl-2,6-dimethyl-1,4-dihydropyridine-3,5-dicarboxylates are well known as a class of calcium channel blockers [2,3]. Substituents at positions 3 and 5 of 4-aryl-2,6-dimethyl-1,4-dihydropyridine-3,5-dicarboxylates have crucial influence on biological activities and chemical properties such as hydrolysis, and transesterification [4]. Thus, alkyl esters at positions 3 and 5 of 1,4-DHP are exceptionally stable upon treatment with nucleophilic reagents due to electronic and steric reasons [5]. In the case of ethoxycarbonylmethyl 1,4-dihydropyridine-3-carboxylates, alkaline and enzyme-catalysed hydrolysis takes place only at the more remote from the 1,4-DHP cycle ester groups forming the corresponding carboxymethyl esters because of steric and electronic factors [6,7]. The antiviral activity of 1,4-DHP-3,5-bis(alkoxymethylcarboxylates) and a pronounced synergism with the anti-cancer drug 5-FU have been reported recently [8,9]. Studies of carbamoylmethyl 1,4-DHP-3-carboxylates are rather limited and fragmented. There is one reported synthesis of phenylcarbamoylmethyl ester of 1,4-DHP via a three component Hantzsch-type condensation [10,11]. However, this approach is avoided since the commercially unavailable carbamoylmethylacetoacetates would require synthesis from diketene. Another method involves hydrolysis of the ethoxycarbonylmethyl ester of 1,4-DHP followed by activation of the resulting acid and amidation reaction with an amine [12]. Previously our laboratory reported that the 3-ethoxycarbonylmethyl ester containing 1,4-DHP derivatives together with removal of a protecting group using methylamine readily formed methylcarbamoylmethyl esters which possess calcium level controlling activities [13]. Amidation of the appropriate acids through activated intermediates with secondary amines remains the only option leading to tertiary carbamoylmethyl esters.

During the last decade a noticeable improvement for direct aminolysis of acetates or benzoates has been achieved by using amine-based bases as catalysts: imidazole [14], TEA [15], 2-pyridone [16], TBD [17], DBU [18], and others. It is worth mentioning that primary amines have recently been reported for aminolysis of alkoxycarbonylmethyl ester of asiatic acid [19], substituted piperidines [20] and substituted alkoxymethyl oxycarbamates [21], however no catalytic methods to promote this slow reaction have been found up to now.

In the case of 1,4-DHPs the aminolysis is more preferential, versatile, and faster method since the starting materials are easily obtained via Hantzsch synthesis. From the synthetic point of view elaboration of synthetic procedures for convenient construction of the desired carbamoylmethyl esters of 4-aryl-2,6-dimethyl-1,4-dihydropyridine-3,5-dicarboxylates through direct aminolysis is the main objective of this work. In this paper, we report our studies on aminolysis of the ethoxycarbonylmethyl ester of 1,4-dihydropyridines **3a**–**d** with primary and secondary alkyl and cyclic amines.

## 2. Results and Discussion

**Scheme 1:**
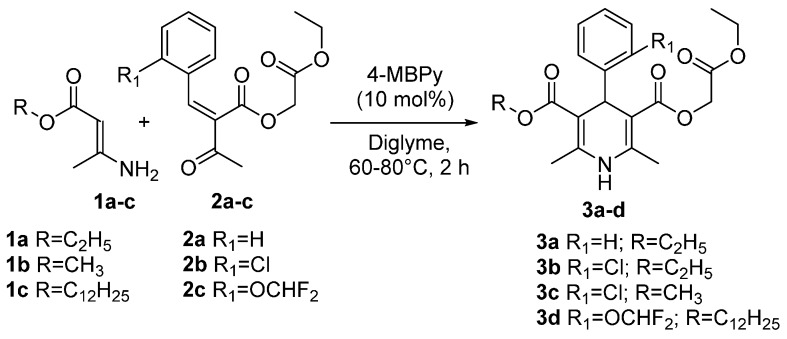
Synthesis of ethoxycarbonylmethyl esters of 1,4-dihydropyridines **3a**–**d**.

The starting ethoxycarbonylmethyl esters of 1,4-dihydropyridines **3a**–**d** (Scheme 1) were prepared via a two-component Hantzsch type cyclisation of the appropriate ethoxycarbonylmethyl 3-oxo-(1-arylmethylidene)butyrate [13] **2a**–**c** and 3-aminobut-2-enoic acid ester [22] **1a**–**c** utilising by using diglyme as solvent and 1-butyl-4-methylpyridinium chloride (4-MBPy) as catalyst. The reactions were carried out at 60–80 °C for two hours giving ethoxycarbonylmethyl esters of 1,4-dihydropyridines **3a**–**d** in 33%–75% yields in analogy with already reported methodologies [23]. Synthesis of 1,4-DHPs **3b**–**d** was performed based on the fact that these motifs are common structural scaffolds in drug molecules.

**Scheme 2:**
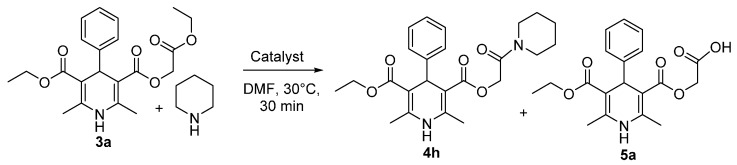
Screening of various catalysts for aminolysis using 1,4-DHP **3a** and piperidine as model substrates.

Recently a non-catalytic direct aminolysis of 3-ethoxycarbonylmethyl ester containing 1,4-DHP derivatives with methylamine leading to the corresponding methylcarbamoylmethyl esters in 48 h was described [13]. To explore the scope and limitation of this reaction a variety of primary and secondary alkylamines and cyclic amines were reacted with 1,4-DHPs **3a**–**d**. Primary aminolysis screening of ethoxycarbonylmethyl ester of 1,4-dihydropyridine **3a** with piperidine as a model substrate, utilising by using *N*-containing bases, such as TEA, Pyridine, DMAP, DBU, DBN, Imidazole, TMG, TBD and MTBD as catalysts was performed and the results are summarised in Scheme 2 and Table 1.

Screening reactions were carried out with 1 equiv. of 1,4-DHP **3a**, 3 equiv. of piperidine and 20 mol % of the appropriate catalyst, at 30 °C for thirty minutes in DMF and the reaction rates were measured with LC-MS technique. The amount of DMF used for dissolution of 1.5 mmol of 1,4-DHP **3a** was 1 mL. It was found that only the bicyclic guanidine base TBD catalysed aminolysis reaction proceeded efficiently (Table 1, entry 9). For the rest of the cases where other bases were used as catalysts (Table 1, entries 2–8 and 10) and in the absence of a catalyst (Table 1, entry 1) no aminolysis reaction proceeded, as only trace amounts of carboxymethyl derivative **5a** were found.

**Table 1:** Screening of an efficient catalyst for aminolysis reactions of ethoxycarbonylmethyl ester of 1,4-dihydropyridine **3a** with piperidine.

Entry	Catalyst	Time, h	Conversion Rate of 3a, % *	Selectivity to 4 h, % *	Selectivity to 5a, % *
1	-	0.5	<1	-	>99
2	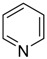	0.5	<1	-	>99
	(pyridine)				
3	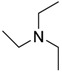	0.5	<1	-	>99
	(TEA)				
4	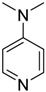	0.5	<1	-	>99
	(DMAP)				
5	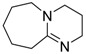	0.5	<1	-	>99
	(DBU)				
6	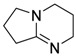	0.5	<1	-	>99
	(DBN)				
7	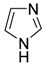	0.5	<1	-	>99
	(imidazole)				
8	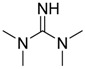	0.5	<1	-	>99
	(TMG)				
9	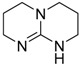	0.5	85	97	3
	(TBD)				
10	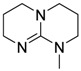	0.5	<1	-	>99
	(MTBD)				

* determined with LC-MS technique.

Such a lack of reactivity of other catalysts in the aminolysis reaction is perhaps due to their lower basicity compared to TBD [24,25], steric hindrance and low nucleophilicity. For guanidine-catalysed reactions an interplay between basicity and nucleophilicity of the catalyst was already considered as crucial [26].

As TBD possessed the highest catalytic activity to provide amide **4h**, this base was selected for further experiments. TBD is an efficient guanidine-based bifunctional catalyst, which has found applications in promotion of various reactions including aminolysis of benzoates and methylphenyl acetates [17]. Screening of three TBD loadings (5, 10 and 20 mol %) for aminolysis reactions of the ethoxycarbonylmethyl ester of 1,4-dihydropyridine **3a** with piperidine have been evaluated (Table 2, entries 1–3). Using 5 mol % of TBD (Table 2, entry 3) the conversion of 1,4-DHP **3a** led mainly to the formation of carboxymethyl derivative **5a** with minor formation of amide **4h**. The catalytic activity of TBD was remarkable at 10 mol % loading, however after 4 h the reaction was still incomplete, with formation of carboxymethyl derivative **5a** as by-product (Table 2, entry 2). Increasing the catalyst loading from 10 to 20 mol % secured a complete transformation of ester **3a** into amide **4h**, with almost no formation of carboxymethyl derivative **5a** (Table 2, entry 1). Catalyst loading was selected as 20 mol % as it was used in the screening experiments.

**Table 2:** The effect of solvent, temperature and TBD amount on aminolysis reactions of ethoxycarbonylmethyl ester of 1,4-dihydropyridine **3a** with piperidine.

Entry	TBD, mol %	Time, h	Temp, °C	Solvent	Conversion Rate of 3a, % *	Selectivity to 4 h, % *	Selectivity to 5a, % *
1	20	4	30	DMF	98	99	1
2	10	4	30	DMF	54	88	12
3	5	4	30	DMF	41	27	73
4	20	4	4	DMF	77	97	3
5	20	0.5	70	DMF	98	89	11
6	20	4	30	THF	94	96	4
7	20	4	30	MeCN	97	79	21
8	20	4	30	CH_2_Cl_2_	79	61	39
9	20	4	30	Dioxane	65	56	44
10	20	4	30	MeOH	99	74	26

* determined with LC-MS technique.

Further experiments have revealed that the aminolysis at 70 °C was much faster together with formation of carboxymethyl derivative **5a** but decreasing the temperature to 4 °C slowed the reaction down (Table 2, entries 4 and 5). Therefore, performing the reaction at 30 °C was found to be optimal and this temperature was used in further experiments.

Altering the reaction media from DMF (Table 2, entry 1) to THF led to rather similar results (Table 2, entry 6). Selectivity of the reaction was changed considerably when acetonitrile, dichloromethane, dioxane or methanol were used as solvents. Aminolysis reaction, with the same TBD and piperidine loading performed in dichloromethane or dioxane led to extensive formation of carboxymethyl derivative **5a** as by-product along with recovered starting material (Table 2, entries 7–9). Performing aminolysis in methanol led to a complete disappearance of the starting ester **3a** with formation of a mixture of carboxymethyl derivative **5a** and amide **4h** (Table 2, entry 10).

**Scheme 3:**
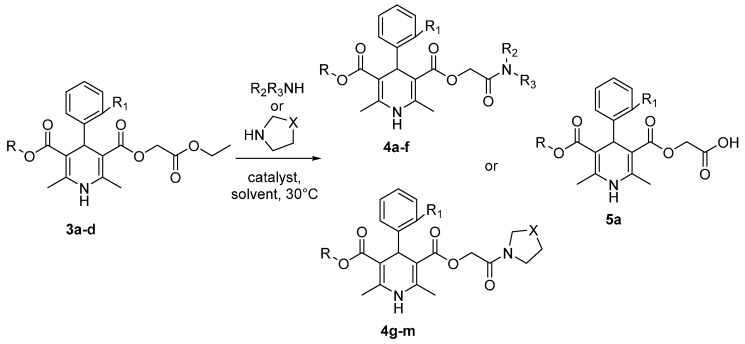
Aminolysis of ethoxycarbonylmethyl esters of 1,4-dihydropyridines **3a**–**d**.

Four 1,4-dihydropyridine derivatives **3a**–**d** having an ethoxycarbonylmethyl ester group at position 3 (or 5) and differing in the substituents at position 4 and other ester position 5 (or 3) were selected for aminolysis with various primary, secondary alkyl and cyclic amines such as 1-propyl-, 2-propyl-, 1-butyl-, diethyl-, diisopropyl-, diphenyl-, *N*-methylbutyl-, *N*-methyloctylamine, pyrrolidine, piperidine, morpholine, thiomorpholine and 1-naphthylamine (Scheme 3).

Next, we explored the influence of TBD on the aminolysis reactions of 1,4-DHP **3a** with primary amines (Table 3, entries 1–7). TBD catalysed aminolysis of 1,4-DHP **3a** using 3 fold excess of 1-propylamine and DMF as the solvent (Table 3, entry 1) proceeded smoothly with 78% yield in 2 h at 30 °C. In the absence of a catalyst in 2 h aminolysis of 1,4-DHP **3a** with 1-propylamine at the same reaction conditions practically does not occur as only 2% of amide **4a** was detected (Table 3, entry 2). The reaction of ester **3a** was performed by using 2-propylamine as also the solvent, that is in the presence of 15 fold excess of nucleophile for 4 h at 30 °C in 76% yield (Table 3, entry 5), and by adding the amine only in a reduced amount (3 equiv.) to DMF the amide **4b** was obtained in 79% yield (Table 3, entry 3). Without adding a catalyst reaction of 1,4-DHP **3a** with 2-propylamine proceeded very slowly in DMF at 30 °C, as only 4% of conversion to amide **4b** was observed in 4 h (Table 3, entry 4), a longer reaction time did not improve this reaction significantly. The influence of TBD on aminolysis of ester **3a** using a 3 fold excess of 1-butylamine (Table 3, entries 6, 7) in DMF was also studied. The usefulness of TBD as a catalyst has been proven also in this case, as almost quantitative conversion of ester **3a** to amide **4c** in the case of TBD catalysed reaction (Table 3, entry 6) was observed compared to only 3% of conversion to **4c** in the case where no catalyst was used (Table 3, entry 7). 

Similarly to the reactions of 1,4-DHP **3a** with primary amines in the presence of TBD, the secondary alkyl and cyclic amines also showed good activity toward 1,4-DHPs **3a**–**d** under the present reaction conditions, thus in three or four hours 75%–97% yields of the target amides **4d**–**m** were reached (Scheme 3, Table 3, entries 8, 11–18, 20–23). When TBD was applied to the reaction of ester **3a** with diethylamine in DMF for 4 h at 30 °C, amide **4d** was obtained in 75% yield (Table 3, entry 8). No aminolysis reaction was observed when ester **3a** was treated with the bulkier diisopropylamine or diphenylamine perhaps due to steric reasons (Table 3, entries 9, 10). However, ethoxycarbonylmethyl ester **3a** was hydrolysed to carboxymethyl derivative **5a** in 70% with diisopropylamine (Table 3, entry 9) as this base is considerably stronger than diphenylamine. In the case of diphenylamine no hydrolysis of ester **3a** was observed (Table 3, entry 10). Reactions of 1,4-DHP **3a** with unsymmetrical secondary amines—*N*-methylbutylamine or *N*-methyloctylamine (Table 3, entries 11 and 12) also proceeded smoothly with high yields in the presence of TBD in DMF for 4 h at 30 °C. Reactions of **3a**–**d** with piperidine, morpholine, pyrrolidine and thiomorpholine were performed in DMF for three or four hours at 30 °C in the presence of TBD (Table 3, entries 13, 14, 16 and 18, 20–22). No aminolysis of ester **3a** with the sterically bulky 1-naphthylamine was observed (Table 3, entry 19). Alternatively, reactions of esters **3a**,**d** with piperidine and morpholine can be performed where nucleophiles were also used as solvents (Table 3, entries 15, 17 and 23), however in this case a 15 fold excess of amine should be used. When aminolysis reactions were performed in DMF the amines were used in a 3-fold excess only.

It was also shown in the examples of aminolysis of 1,4-dihydropyridines **3b**–**d** having substituted aromatic ring at position 4 (Table 3, entries 20–23) and 1,4-dihydropyridines **3c,d** (Table 3, entries 21–23) having other ester moieties on the other side of 1,4-DHP ring that there is no significant influence of these substituents on the aminolysis reaction with cyclic amines.

Altering the reaction media had no significant influence on the yields of the reactions (Table 3, entries 3 *vs.* 5, 14 *vs.* 15, 16 *vs.* 17 and 22 *vs.* 23). It should be admitted that aminolysis reactions of 1,4-DHP **3a**–**d** with secondary amines in the absence of TBD did not proceed at all.

**Table 3:** Aminolysis of ethoxycarbonylmethyl esters of 1,4-dihydropyridines **3a**–**d**.

Entry	Amine	R	R^1^	Substrate	Solvent	Catalyst	R^2^	R^3^	X	Time, h	Product	Yield, %
1	1-Propylamine	Et	H	3a	DMF	TBD	n-Pr	H	-	2	4a	78
2	1-Propylamine	Et	H	3a	DMF	**-**	n-Pr	H	-	2	4a	2 *
3	2-Propylamine	Et	H	3a	DMF	TBD	i-Pr	H	-	4	4b	79
4	2-Propylamine	Et	H	3a	DMF	-	i-Pr	H	-	4	4b	4 *
5	2-Propylamine	Et	H	3a	2-Propylamine	TBD	i-Pr	H	-	4	4b	76
6	1-Butylamine	Et	H	3a	DMF	TBD	n-Bu	H	-	4	4c	98
7	1-Butylamine	Et	H	3a	DMF	-	n-Bu	H	-	4	4c	3 ^*^
8	Diethylamine	Et	H	3a	DMF	TBD	Et	Et	-	4	4d	75
9	Diisopropylamine	Et	H	3a	DMF	TBD	-	-	-	4	5a	70
10	Diphenylamine	Et	H	3a	DMF	TBD	-	-	-	4	-	-
11	*N*-Methylbutylamine	Et	H	3a	DMF	TBD	Me	n-Bu	-	4	4e	89
12	*N*-Methyloctylamine	Et	H	3a	DMF	TBD	Me	n-C_8_H_17_	-	4	4f	87
13	Pyrrolidine	Et	H	3a	DMF	TBD	-	-	CH_2_	3	4g	94
14	Piperidine	Et	H	3a	DMF	TBD	-	-	CH_2_CH_2_	3	4h	89
15	Piperidine	Et	H	3a	Piperidine	TBD	-	-	CH_2_CH_2_	3	4h	85
16	Morpholine	Et	H	3a	DMF	TBD	-	-	CH_2_O	4	4i	97
17	Morpholine	Et	H	3a	Morpholine	TBD	-	-	CH_2_O	4	4i	94
18	Thiomorpholine	Et	H	3a	DMF	TBD	-	-	CH_2_S	3	4j	80
19	1-Naphthylamine	Et	H	3a	DMF	TBD	-	-	-	4	-	-
20	Thiomorpholine	Et	Cl	3b	DMF	TBD	-	-	CH_2_S	3	4k	85
21	Pyrrolidine	Me	Cl	3c	DMF	TBD	-	-	CH_2_	3	4l	96
22	Piperidine	C_12_H_25_	OCHF_2_	3d	DMF	TBD	-	-	CH_2_CH_2_	3	4m	87
23	Piperidine	C_12_H_25_	OCHF_2_	3d	Piperidine	TBD	-	-	CH_2_CH_2_	3	4m	87

* determined by HPLC.

Structures of all newly synthesised 1,4-DHPs were established and confirmed by ^1^H-NMR, ^13^C-NMR, MS, IR and elemental analysis data. The IR spectra of obtained compounds showed characteristic 1,4-DHPs absorption bands. Thus, N-H and C=O absorption bands were present at frequencies 3398–3200 cm^−1^ and 1750–1621 cm^−1^, respectively. The presence of C=O absorbance at ~1740 cm^−1^ of the more remote carbonyl group from the 1,4-DHP cycle of esters **3a**–**d** and acid **5a** was observed, while for amides **4a**–**m** this absorbance was shifted to lower frequencies (~1700 cm^−1^) and merged with C=O signals of the conjugated β-aminovinylcarbonyl system of 1,4-DHP. Molecular weights of compounds measured by LC-MS technique were in good agreement with the calculated values for all the compounds. The ^1^H-NMR spectra obtained in both solvents CDCl_3_ and DMSO-*d*_6_ of all newly synthesised 1,4-DHP esters **3a**–**d**, amides **4a**–**m** and carboxymethyl derivative **5a** have shown that the methylene group protons (COOCH_2_CO) at the position 3 (or 5) have appeared as AB-systems at δ interval between 4.10 and 4.86 with a coupling constant over 14 Hz. Chemical shifts and coupling constant values were dependent on the substituents of 1,4-DHP derivatives **3a**–**d**, **4a**–**m**, **5a** and the solvents. It should be underlined that some of the signals in NMR spectra of carbamoylmethyl ester of 1,4-dihydropyridines **4e** and **4f** having unsymmetrical tertiary carbamoyl group were split due to nitrogen inversion of amide and slow N-C(O) bond rotation. Thus, the proton signals in ^1^H-NMR spectra of AB-systems of methylene group and N-CH_3_ group were split into two components with ratio—0.45:0.55. The carbon signals in ^13^C-NMR spectra were also duplicated for some alkyl carbons of both carbonyl group carbons at the carbamoylmethyl ester of 1,4-DHPs **4e** and **4f**. Similar observations were found for *N*-acyl oxaziridine systems confirming the existence of two independent stereodynamic processes: nitrogen inversion and rotation about the N-C(O) bond, both processes were determined to be slow on the NMR timescale [27]. The interpretation of the conformational information for the Me-N-C(O)-CH_2_ fragment of the carbamoylmethyl ester **4f** was based on ^1^H-^1^H NOESY spectra. The observed cross peak between N-CH_3_ (δ: 2.84) from the major conformer and methylene group (COOCH_2_CO) signals showed that these groups were close in space. Thus, the signal of N-CH_3_ (δ: 2.84) belongs to the *cis*-isomer (55%). The observed cross peak between N-CH_2_- (δ: 3.12) from the minor conformer and methylene group (COOCH_2_CO) signals showed that these groups were close in space. Thus, the signal of N-CH_2_- (δ: 3.12) belongs to the *trans*-isomer (45%).

A potential mechanism for the aminolysis of methyl benzoate and methyl phenylacetate in the presence of TBD has been proposed [17], which includes TBD reaction with ester followed by proton transfer from the protonated nitrogen of TBD with further formation of the corresponding TBD amide and alcohol from the ester moiety. Finally, regeneration of free TBD resulted in the formation of amides. Theoretical studies of aminolysis of methyl acetate catalysed by TBD performed by Jin *et al* have indicated a stepwise mechanism involving tetrahedral intermediates through hydrogen bonding of TBD [28].

## 3. Experimental Section

### 3.1. General Information

All reagents were purchased from Acros Organics (Geel, Belgium), Sigma-Aldrich (St. Louis, MO, USA), Alfa Aesar (Lancashire, UK), or Merck KGaA (Darmstadt, Germany) and used without further purification. TLC was performed on silica gel 60 F_254_ aluminium sheets 20 × 20 cm (Merck KGaA, Darmstadt, Germany). ^1^H-NMR (400 MHz) and ^13^C-NMR (100.56 MHz) spectra were recorded with a Varian Mercury BB spectrometer (Agilent, Santa Clara, CA, USA). The coupling constants are expressed in Hertz (Hz). The chemical shifts of the hydrogen and carbon atoms are presented in parts per million (ppm) and referred to the residual signals of the non-deuterated CDCl_3_ (δ: 7.26) or partially deuterated DMSO-*d*_6_ (δ: 2.50) solvent for ^1^H-NMR spectra and CDCl_3_ (δ: 77.0) or DMSO-*d*_6_ (δ: 39.5) solvent for ^13^C-NMR, respectively. Multiplicities are abbreviated as s = singlet; d = doublet; t = triplet; m = multiplet; br = broad; dd = double doublet; dt = double triplet; td = triple doublet; tt = triple triplet; ddd = double double doublet. Mass spectral data were determined on an Acquity UPLC system (Waters, Milford, MA, USA) connected to a Waters SQ Detector-2 operating in the ESI positive or negative ion mode on a Waters Acquity UPLC^®^ BEH C18 column (1.7 µm, 2.1 × 50 mm, using a gradient elution with acetonitrile (0.01% trifluoroacetic acid) in water (0.01% trifluoroacetic acid) at a flow rate of 0.5 mL/min. LC-MS data were recorded with a Waters MassLynx 4.1 chromatography data system. The conversions of the reactions were analysed by HPLC on Waters Alliance 2695 system and Waters 2485 UV/Vis detector equipped with Alltima ODS-2 column (5 µm, 4.6 × 150 mm, Grace, Columbia, MD, USA) using a gradient elution with methanol/water (*v*/*v*), at a flow rate of 1 mL/min. Peak areas were determined electronically with Waters Empower 2 chromatography data system. Melting points (m.p.) of the synthesised compounds were determined on an OptiMelt (SRS Stanford Research Systems, Sunnyvale, CA, USA). Infrared spectra were recorded with a Prestige-21 FTIR spectrometer (Shimadzu, Kyoto, Japan). Elemental analyses were determined on an Elemental Combustion System ECS 4010 (Costech Instruments, Pioltello, Italy).

### 3.2. General Procedure for the Synthesis of Ethoxycarbonylmethyl Esters of 1,4-Dihydropyridines ***3a**–**d***

A mixture of the appropriate 3-aminobut-2-enoic acid ester **1a**–**c** (3 mmol), ethoxycarbonylmethyl 3-oxo-(1-arylmethylidene)butyrate **2a**–**c** (3 mmol) and 1-butyl-4-methylpyridinium chloride (56 mg, 10 mol %) in diglyme (15 mL) was heated for two hours at 60–80 °C. After cooling the resulting mixture was poured on crushed ice, after which the precipitate was filtered off, washed with water and crystallised from methanol giving 1,4-DHPs **3a**–**d** as pale yellow powders.

*3-(2-Ethoxy-2-oxoethyl) 5-ethyl 2,6-dimethyl-4-phenyl-1,4-dihydropyridine-3,5-dicarboxylate* (**3a**). Yield 51%; light yellow crystals; m.p. 113 °C. IR (film) 3351, 3250, 1743, 1701, 1654, 1624 cm^−1^. ^1^H-NMR (CDCl_3_) 7.30–7.27 (m, 2H), 7.22–7.17 (m, 2H), 7.14–7.09 (m, 1H), 6.01 (br s, 1H), 5.05 (s, 1H), 4.61 and 4.53 (AB-system, *J =* 15.7, 2H), 4.18 (q, *J =* 7.2, 2H), 4.12–4.04 (m, 2H), 2.33 (s, 3H), 2.32 (s, 3H), 1.23 (t, *J =* 7.4, 3H), 1.21 (t, *J =* 7.2, 3H); ^13^C-NMR (CDCl_3_) 168.6, 167.6, 167.0, 147.6, 145.9, 143.9, 128.0, 126.3, 104.7, 102.9, 61.3, 60.6, 59.9, 39.5, 19.9, 19.5, 14.4, 14.2; MS (+ESI) *m*/*z* (relative intensity) 388 ([M + H]^+^, 50). Anal. Calc. for C_21_H_25_NO_6_: C, 65.10; H, 6.50; N, 3.62; found: C, 64.89; H, 6.54; N, 3.59.

*3-(2-Ethoxy-2-oxoethyl) 5-ethyl 4-(2-chlorophenyl)-2,6-dimethyl-1,4-dihydropyridine-3,5-dicarboxylate* (**3b**). Yield 33%; white powder; m.p. 111 °C. IR (film) 3359, 3248, 1741, 1700, 1649, 1621 cm^−1^. ^1^H-NMR (DMSO-*d*_6_) 8.95 (br s, 1H), 7.34–7.31 (m, 1H), 7.24–7.18 (m, 2H), 7.12–7.07 (m, 1H), 5.29 (s, 1H), 4.54 and 4.48 (AB-system, *J =* 15.6, 2H), 4.05 (q, *J =* 7.0, 2H), 3.95 (dq, *J =* 7.0 and *J =* 2.0, 2H), 2.25 (s, 6H), 1.12 (t, *J =* 7.0, 3H), 1.09 (t, *J =* 7.0, 3H); ^13^C-NMR (DMSO-*d*_6_) 168.0. 166.7, 166.2, 146.8, 146.0, 145.2, 131.3, 131.1, 128.9, 127.5, 127.2, 102.3, 100.5, 60.5, 60.0, 59.0, 36.6, 18.2, 18.1, 14.2, 13.9; MS (+ESI) *m*/*z* (relative intensity) 422 ([M + H]^+^, 100). Anal. Calc. for C_21_H_24_ClNO_6_: C, 59.79; H, 5.73; N, 3.32; found: C, 59.63; H, 5.69; N, 3.23.

*3-(2-Ethoxy-2-oxoethyl) 5-methyl 4-(2-chlorophenyl)-2,6-dimethyl-1,4-dihydropyridine-3,5-dicarboxylate* (**3c**). Yield 47%; white powder; m.p. 95 °C. IR (film) 3355, 3200, 1743, 1700, 1645 cm^−1^. ^1^H-NMR (DMSO-*d*_6_) 8.98 (s, 1H), 7.33–7.31 (m, 1H), 7.24–7.20 (m, 2H), 7.11–7.08 (m, 1H), 5.30 (s, 1H), 4.54 and 4.48 (AB-system, *J =* 16.0, 2H), 4.04 (q, *J =* 8.0 Hz, 2H), 3.49 (s, 3H), 2.26 (s, 3H), 2.24 (s, 3H), 1.11 (t, *J =* 8.0, 3H); ^13^C-NMR (DMSO-*d*_6_) 168.4, 167.6, 166.6, 147.4, 146.5, 145.7, 131.7, 131.3, 129.4, 128.0, 127.7, 102.6, 101.1, 60.9, 60.4, 50.9, 37.0, 18.7, 18.4, 14.3; MS (+ESI) *m*/*z* (relative intensity) 408 ([M + H]^+^, 40). Anal. Calc. for C_20_H_22_ClNO_6_: C, 58.90; H, 5.44; N, 3.43; found: C, 58.68; H, 5.35; N, 3.33.

*3-Dodecyl 5-(2-ethoxy-2-oxoethyl) 4-(2-(difluoromethoxy)phenyl)-2,6-dimethyl-1,4-dihydropyridine-3,5-dicarboxylate* (**3d**). Yield 75%; white powder; m.p. 76 °C. IR (film) 3354, 3103, 1742, 1698, 1650 cm^−1^. ^1^H-NMR (CDCl_3_) 7.37 (dd, *J =* 7.8 Hz and *J =* 1.9, 1H), 7.15–7.09 (m, 1H), 7.08–7.02 (m, 1H), 6.96 (d, *J =* 7.8, 1H), 6.47 (dd, *J =* 73.9 Hz and *J =* 76.7, 1H), 6.03 (br s, 1H), 5.31 (s, 1H), 4.56 and 4.50 (AB-system, *J =* 15.5, 2H), 4.14 (q, *J =* 7.2, 2H), 4.02–3.92 (m, 2H), 2.30 (s, 3H), 2.28 (s, 3H), 1.60–1.51 (m, 2H), 1.32–1.22 (m, 18H) overlap, 1.20 (t, *J =* 7.2, 3H) overlap, 0.88 (t, *J =* 7.1, 3H); ^13^C-NMR (CDCl_3_) 167.7, 167.6, 167.0, 149.7, 146.1, 144.2, 138.5, 132.0, 127.7, 125.0, 118.0, 117.8 (t, *J =* 248 Hz), 103.5, 101.7, 64.2, 61.3, 60.4, 36.0, 32.0, 29.9, 29.8, 29.7, 29.6, 29.5, 29.4, 28.7, 26.1, 22.8, 19.8, 19.5, 14.2, 14.1; MS (+ESI) *m*/*z* (relative intensity) 594 ([M + H]^+^, 60). Anal. Calc. for C_32_H_45_F_2_NO_7_: C, 64.74; H, 7.64; N, 2.36; found: C, 64.75; H, 7.66; N, 2.29.

### 3.3. General Procedure for the Aminolysis of Ethoxycarbonylmethyl Esters of 1,4-Dihydropyridines ***3a**–**d*** in DMF

To the mixture of 1,4-DHP **3a**–**d** (1 equiv.) and the appropriate amine (3 equiv.) in DMF (1 mL was used per 3 mmol of the corresponding 1,4-DHP **3a**–**d**) TBD (20 mol %) was added at rt. After being stirred at 30 °C for 2, 3, or 4 h, the reaction mixture was concentrated under reduced pressure. The residue was triturated with water, filtered off and crystallised from ethanol giving amides **4a**–**m**.

*3-Ethyl 5-(2-oxo-2-(propylamino)ethyl) 2,6-dimethyl-4-phenyl-1,4-dihydropyridine-3,5-dicarboxylate* (**4a**). Yield 78%; white powder; m.p. 125–127 °C. IR (film) 3398, 3305, 3229, 1702, 1658, 1623 cm^−1^. ^1^H-NMR (CDCl_3_) 7.20–7.14 (m, 4H) overlap with CHCl_3_, 7.18–7.12 (m, 1H), 6.55–6.35 (m, 1H), 5.26 (br s, 1H), 4.98 (s, 1H), 4.86 and 4.14 (AB-system, *J =* 15.0, 2H) overlap, 4.19–4.04 (m, 2H) overlap, 2.89–2.88 (m, 1H), 2.85–2.74 (m, 1H), 2.38 (s, 3H), 2.24 (s, 3H), 1.25 (t, *J =* 6.8, 3H) overlap, 1.28–1.16 (m, 2H) overlap, 0.73 (t, *J =* 7.2, 3H); ^13^C-NMR (CDCl_3_) 168.0, 167.6, 165.7, 147.9, 143.6, 128.6, 127.9, 126.7, 104.9, 101.4, 60.0, 40.7, 39.6, 19.5, 14.4, 11.3; MS (-ESI) *m*/*z* (relative intensity) 399 ([M − H]^−^, 50). Anal. Calc. for C_22_H_28_N_2_O_5_: C, 65.98; H, 7.05; N, 7.00; found: C, 65.79; H, 7.03; N, 6.87.

*3-Ethyl 5-(2-(isopropylamino)-2-oxoethyl) 2,6-dimethyl-4-phenyl-1,4-dihydropyridine-3,5-dicarboxylate* (**4b**). Yield 79%; light yellow powder; m.p. 116 °C. IR (film) 3305, 3229, 1699, 1648 cm^−1^. ^1^H-NMR (CDCl_3_) 7.29–7.25 (m, 2H) overlap with CHCl_3_, 7.24–7.19 (m, 2H), 7.17–7.11 (m, 1H), 6.17 (br s, 1H), 5.33–5.26 (m, 1H), 4.95 (s, 1H), 4.72 and 4.19 (AB-system, *J =* 15.6, 2H), 4.15–4.03 (m, 2H), 4.00–3.89 (m, 1H), 2.37 (s, 3H), 2.24 (s, 3H), 1.23 (t, *J =* 7.5, 3H), 0.94 (d, *J =* 6.5, 3H), 0.83 (d, *J =* 6.5, 3H); ^13^C-NMR (CDCl_3_) 167.5, 167.0, 165.6, 147.8, 147.6, 143.6, 128.5, 127.8, 127.5, 126.7, 104.7, 101.2, 62.1, 59.8, 40.7, 39.3, 22.4, 22.2, 19.4, 19.3, 14.3; MS (+ESI) *m*/*z* (relative intensity) 401 ([M + H]^+^, 30). Anal. Calc. for C_22_H_28_N_2_O_5_: C, 65.98; H, 7.05; N, 6.99; found: C, 65.85; H, 6.97; N, 6.97.

*3-(2-(Butylamino)-2-oxoethyl) 5-ethyl 2,6-dimethyl-4-phenyl-1,4-dihydropyridine-3,5-dicarboxylate* (**4c**). Yield 98%; light yellow powder; m.p. 124 °C. IR (film) 3397, 3315, 3228, 1685, 1645 cm^−1^. ^1^H-NMR (CDCl_3_) 7.29–7.25 (m, 2H) overlap with CHCl_3_, 7.24–7.18 (m, 2H), 7.14–7.09 (m, 1H), 6.03 (br s, 1H), 5.25–5.18 (m, 1H), 4.95 (s, 1H), 4.82 and 4.10 (AB-system, *J =* 15.8, 2H) overlap, 4.13–4.03 (m, 2H) overlap, 2.98–2.88 (m, 1H), 2.85–2.75 (m, 1H), 2.36 (s, 3H), 2.22 (s, 3H), 1.22 (t, *J =* 7.2, 3H), 1.18–1.08 (m, 4H), 0.80 (t, *J =* 6.8, 3H); ^13^C-NMR (CDCl_3_) 168.0, 167.5, 165.4, 148.0, 147.9, 143.6, 128.4, 127.7, 126.6, 104.7, 101.1, 61.7, 59.9, 39.4, 38.7, 31.4, 19.9, 19.3, 14.3, 13.7; MS (+ESI) *m*/*z* (relative intensity) 415 ([M + H]^+^, 30). Anal. Calc. for C_23_H_30_N_2_O_5_: C 66.65; H 7.30; N 6.76; found: C, 66.38; H, 7.21; N, 6.90.

*3-(2-(Diethylamino)-2-oxoethyl) 5-ethyl 2,6-dimethyl-4-phenyl-1,4-dihydropyridine-3,5-dicarboxylate* (**4d**). Yield 75%; light yellow crystals; m.p. 181 °C. IR (film) 3290, 3222, 1699, 1640 cm^−1^. ^1^H-NMR (CDCl_3_) 7.30–7.27 (m, 2H), 7.21–7.16 (m, 2H), 7.12–7.08 (m, 1H), 6.26 (br s, 1H), 5.05 (s, 1H), 4.76 and 4.58 (AB-system, *J =* 14.2, 2H), 4.11–3.99 (m, 2H), 3.46–3.31 (m, 2H), 3.19 (q, *J =* 7.0, 2H), 2.36 (s, 3H), 2.34 (s, 3H), 1.19 (t, *J =* 7.0, 3H) overlap, 1.18–1.11 (m, 6H) overlap; ^13^C-NMR (CDCl_3_) 167.6, 167.3, 166.5, 148.2, 146.1, 144.1, 128.1, 127.7, 125.9, 104.1, 102.4, 60.7, 59.5, 41.0, 40.4, 39.6, 19.4, 19.0, 14.2, 14.1, 12.9; MS (+ESI) *m*/*z* (relative intensity) 415 ([M + H]^+^, 50). Anal. Calc. for C_23_H_30_N_2_O_5_: C 66.65; H 7.30; N 6.76; found: C, 66.33; H, 7.27; N, 6.68.

*3-(2-(Butyl(methyl)amino)-2-oxoethyl) 5-ethyl 2,6-dimethyl-4-phenyl-1,4-dihydropyridine-3,5-dicarboxylate* (**4e**). Yield 89%; white powder; m.p. 203 °C. IR (film) 3275, 3218, 1695, 1644, 1629 cm^−1^. ^1^H-NMR (DMSO-*d*_6_) 8.87 (br s, 1H), 7.20–7.14 (m, 4H), 7.11–7.07 (m, 1H), overlap, 4.90 (s, 1H), 4.76 and 4.66 (AB-system, *J =* 14.3, 0.90H), 4.71 and 4.63 (AB-system, *J =* 14.5, 1.10H) overlap, 4.07–3.94 (m, 2H), 3.28–3.20 (m, 1H), 3.18–3.13 (m, 1H), 2.84 (s, 1.65H), 2.77 (s, 1.35H), 2.28 (s, 3H) overlap, 2.27 (s, 3H) overlap, 1.52–1.43 (m, 1H), 1.43–1.35 (m, 1H), 1.28–1.18 (m, 2H), 1.14 (t, *J =* 7.0, 3H), 0.88 (dt, *J =* 7.4 and *J =* 2.7, 3H); ^13^C-NMR (CDCl_3_) 167.5, 167.2, 167.1, 167.0, 166.9, 148.1, 148.0, 146.1, 146.0, 144.0, 143.9, 128.0, 127.7, 125.9, 104.3, 104.2, 102.3, 101.4, 61.0, 60.6, 59.5, 48.6, 47.8, 39.6, 33.9, 33.4, 30.3, 29.2, 20.0, 19.9, 19.6, 19.5, 19.0, 14.2, 13.8, 13.7. MS (−ESI) *m/z* (relative intensity) 427 ([M − H]^−^, 100). Anal. Calc. for C_24_H_32_N_2_O_5_: C, 67.27; H, 7.53; N, 6.54; found: C, 66.99; H, 7.48; N, 6.60.

*3-Ethyl 5-(2-(methyl)(octyl)amino)-2-oxoethyl) 2,6-dimethyl-4-phenyl-1,4-dihydropyridine-3,5-dicarboxylate* (**4f**). Yield 87%; white powder; m.p. 145–147 °C. IR (film) 3283, 3225, 1699, 1644, 1630 cm^−1^. ^1^H-NMR (DMSO-*d*_6_) 8.87 (br s, 1H), 7.21–7.15 (m, 4H), 7.11–7.06 (m, 1H), 4.91 (s, 1H), 4.75 and 4.65 (AB-system, *J =* 14.3, 0.90H) overlap, 4.71 and 4.64 (AB-system, *J =* 14.5, 1.10H) overlap, 4.05–3.94 (m, 2H), 3.25–3.19 (m, 1H), 3.17–3.11 (m, 1H), 2.84 (s, 1.65H), 2.77 (s, 1.35H), 2.29 (s, 3H) overlap, 2.27 (s, 3H) overlap, 1.53–1.45 (m, 1H), 1.44–1.36 (m, 1H), 1.29–1.18 (m, 10H), 1.12 (t, *J =* 7.0, 3H), 0.86 (t, *J =* 7.0, 3H); ^13^C-NMR (DMSO-*d*_6_) 166.9, 166.5, 166.4, 166.2, 166.1, 147.8, 146.1, 146.0, 145.4, 127.9, 127.2, 125.8, 102.0, 101.9, 101.3, 101.2, 60.9, 60.7, 59.0, 47.8, 46.8, 38.5, 33.3, 32.7, 31.3, 31.2, 28.8, 28.7, 28.6, 27.6, 26.6, 26.2, 26.0, 22.1, 22.0, 18.3, 18.2, 14.2, 13.9; MS (−ESI) *m*/*z* (relative intensity) 483 ([M − H]^−^, 100). Anal. Calc. for C_28_H_40_N_2_O_5_: C, 69.39; H, 8.32; N, 5.78; found: C, 69.14; H, 8.38; N, 5.86.

*3-Ethyl 5-(2-oxo-2-(pyrrolidin-1-yl)ethyl) 2,6-dimethyl-4-phenyl-1,4-dihydropyridine-3,5-dicarboxylate* (**4g**). Yield 94%; white crystals; m.p. 202 °C. IR (film) 3283, 3217, 1701, 1643 cm^−1^. ^1^H-NMR (CDCl_3_) 7.27–7.23 (m, 2H) overlap with CHCl_3_, 7.18–7.13 (m, 2H), 7.09–7.05 (m, 1H), 6.89 (br s, 1H), 4.99 (s, 1H), 4.66 and 4.48 (AB-system, *J =* 14.5, 2H), 4.02–3.96 (m, 2H), 3.47 (t, *J =* 6.6, 2H), 3.33–3.26 (m, 1H), 3.24–3.17 (m, 1H), 2.33 (s, 3H), 2.31 (s, 3H), 1.93–1.86 (m, 2H), 1.84–1.77 (m, 2H), 1.16 (t, *J =* 7.0, 3H); ^13^C-NMR (CDCl_3_) 167.7, 167.4, 166.2, 148.4, 146.6, 144.2, 128.3, 127.8, 126.0, 104.4, 102.3, 61.5, 59.6, 46.2, 45.4, 39.8, 26.3, 24.0, 19.6, 19.1, 14.3; MS (+ESI) *m*/*z* (relative intensity) 413 ([M + H]^+^, 40). Anal. Calc. for C_23_H_28_N_2_O_5_: C, 66.97; H, 6.84; N, 6.79; found: C, 66.75; H, 6.82; N, 6.73.

*3-Ethyl 5-(2-oxo-2-(piperidin-1-yl)ethyl) 2,6-dimethyl-4-phenyl-1,4-dihydropyridine-3,5-dicarboxylate* (**4h**). Yield 89%; white crystals; m.p. 203 °C. IR (film) 3286, 3225, 1694, 1645 cm^−1^. ^1^H-NMR (CDCl_3_) 7.29–7.24 (m, 2H) overlap with CHCl_3_, 7.21–7.14 (m, 2H), 7.11–7.06 (m, 1H), 6.30 (br s, 1H), 5.03 (s, 1H), 4.75 and 4.60 (AB-system, *J =* 15.5, 2H), 4.09–3.98 (m, 2H), 3.55–3.49 (m, 2H), 3.23–3.16 (m, 2H), 2.34 (s, 3H), 2.32 (s, 3H), 1.64–1.57 (m, 2H), 1.56–1.45 (m, 4H), 1.18 (t, *J =* 7.2, 3H); ^13^C-NMR (CDCl_3_) 167.7, 167.2, 165.6, 148.1, 146.0, 144.0, 128.2, 128.0, 126.1, 104.5, 102.9, 61.3, 59.7, 45.7, 43.1, 39.7, 26.3, 25.5, 24.5, 19.8, 19.4, 14.4; MS (+ESI) *m*/*z* (relative intensity) 427 ([M + H]^+^, 100). Anal. Calc. for C_24_H_30_N_2_O_5_: C, 67.59; H, 7.09; N, 6.57; found: C, 67.43; H, 7.14; N, 6.40.

*3-Ethyl 5-(2-morpholino-2-oxoethyl) 2,6-dimethyl-4-phenyl-1,4-dihydropyridine-3,5-dicarboxylate* (**4i**). Yield 97%; white crystals; m.p. 184 °C. IR (film) 3293, 3226, 1696, 1647 cm^−1^. ^1^H-NMR (CDCl_3_) 7.31–7.27 (m, 2H) overlap with CHCl_3_, 7.23–7.18 (m, 2H), 7.15–7.10 (m, 1H), 5.70 (br s, 1H), 5.04 (s, 1H), 4.72 and 4.62 (AB-system, *J =* 14.0, 2H), 4.13–4.04 (m, 2H), 3.66–3.51 (m, 6H), 3.25–3.14 (m, 2H), 2.39 (s, 3H), 2.33 (s, 3H), 1.22 (t, *J =* 7.8, 3H); ^13^C-NMR (DMSO-*d*_6_) 166.9, 166.4, 165.5, 147.8, 146.4, 145.3, 127.9, 127.2, 125.9, 102.0, 101.3, 66.0, 65.8, 60.9, 59.1, 44.3, 41.5, 38.5, 18.4, 18.2, 14.2; MS (+ESI) *m*/*z* (relative intensity) 429 ([M + H]^+^, 20). Anal. Calc. for C_23_H_28_N_2_O_6_: C, 64.47; H, 6.59; N, 6.54; found: C, 64.34; H, 6.57; N, 6.47.

*3-Ethyl 5-(2-oxo-2-thiomorpholinoethyl) 2,6-dimethyl-4-phenyl-1,4-dihydropyridine-3,5-dicarboxylate* (**4j**). Yield 80%; white crystals; m.p. 193 °C. IR (film) 3305, 3230, 1700, 1649 cm^−1^. ^1^H-NMR (CDCl_3_) 7.29–7.26 (m, 2H) overlap with CHCl_3_, 7.22–7.18 (m, 2H), 7.14–7.10 (m, 1H), 5.91 (br s, 1H), 5.03 (s, 1H), 4.70 and 4.63 (AB-system, *J =* 14.4, 2H), 4.14–4.01 (m, 2H), 3.88–3.80 (m, 2H), 3.52–3.47 (m, 2H), 2.62–2.57 (m, 2H), 2.55–2.50 (m, 1H), 2.49–2.44 (m, 1H), 2.37 (s, 3H), 2.33 (s, 3H), 1.21 (t, *J =* 7.2, 3H); ^13^C-NMR (CDCl_3_) 167.5, 166.9, 165.8, 147.8, 146.2, 143.3, 128.0, 127.8, 126.1, 104.4, 102.3, 61.2, 59.7, 47.4, 44.5, 39.5, 27.6, 27.2, 19.6, 19.2, 14.2; MS (+ESI) *m*/*z* (relative intensity) 445 ([M + H]^+^, 30). Anal. Calc. for C_23_H_28_N_2_O_5_S: C 62.14; H 6.35; N 6.30; found: C 62.11; H 6.37; N 6.23.

*3-Ethyl 5-(2-oxo-2-thiomorpholinoethyl) 4-(2-chlorophenyl)-2,6-dimethyl-1,4-dihydropyridine-3,5-dicarboxylate* (**4k**). Yield 85%; white crystals; m.p. 179 °C. IR (film) 3298, 3227, 1700, 1654 cm^−1^. ^1^H-NMR (DMSO-*d*_6_) 8.89 (br s, 1H), 7.34–7.31 (dd, *J =* 7.3 Hz and *J =* 1.5, 1H), 7.24–7.17 (m, 2H), 7.12–7.07 (m, 1H), 5.30 (s, 1H), 4.73 and 4.57 (AB-system, *J =* 14.7, 2H), 3.99–3.91 (m, 2H), 3.69–3.62 (m, 2H), 3.57–3.51 (m, 2H), 2.58–2.52 (m, 4H) overlap with DMSO, 2.26 (s, 3H), 2.25 (s, 3H), 1.10 (t, *J =* 7.2, 3H); ^13^C-NMR (DMSO-*d*_6_) 166.8, 166.3, 165.3, 146.2, 146.1, 145.3, 131.3, 131.2, 128.9, 127.5, 127.2, 102.0, 101.1, 61.0, 58.9, 46.7, 43.8, 36.7, 26.8, 26.4, 18.3, 18.1, 14.2; MS (−ESI) *m*/*z* (relative intensity) 477 ([M − H]^−^, 60). Anal. Calc. for C_23_H_27_ClN_2_O_5_S: C, 57.67; H, 5.68; N, 5.85; found: C, 57.63; H, 5.59; N, 5.75.

*3-Methyl 5-(2-oxo-2-(pyrrolidin-1-yl)ethyl) 4-(2-chlorophenyl)-2,6-dimethyl-1,4-dihydropyridine-3,5-dicarboxylate* (**4l**). Yield 96%; white crystals; m.p. 184 °C. IR (film) 3295, 3222, 1700, 1645 cm^−1^. ^1^H-NMR (CDCl_3_) 8.24 (br s, 1H), 7.33–7.37 (m, 1H), 7.17–7.13 (m, 1H), 7.12–7.07 (m, 1H), 6.99–6.94 (m, 1H), 5.36 (s, 1H), 4.83 and 4.29 (AB-system, *J =* 14.9, 2H), 3.51 (s, 3H) overlap, 3.55–3.46 (m, 2H) overlap, 3.39–3.34 (m, 1H), 3.33–3.26 (m, 1H), 2.44 (s, 3H), 2.26 (s, 3H), 2.01–1.93 (m, 2H), 1.90–1.81 (m, 2H); ^13^C-NMR (CDCl_3_) 168.0, 167.6, 166.6, 147.8, 147.2, 145.0, 132.3, 132.1, 128.9, 127.1, 127.0, 103.8, 101.3, 60.6, 50.5, 46.3, 45.4, 37.3, 26.3, 24.0, 18.9, 18.7; MS (+ESI) *m*/*z* (relative intensity) 433 ([M + H]^+^, 30). Anal. Calc. for C_22_H_25_ClN_2_O_5_: C 61.04; H 5.82; N 6.47; found: C, 60.85; H, 5.70; N, 6.39.

*3-Dodecyl 5-(2-oxo-2-(piperidin-1-yl)ethyl) 4-(2-(difluoromethoxy)phenyl)-2,6-dimethyl-1,4-dihydro-pyridine-3,5-dicarboxylate* (**4m**). Yield 87%; light yellow powder; m.p. 95 °C. IR (film) 3300, 3225, 1685, 1654, 1630 cm^−1^. ^1^H-NMR (CDCl_3_) 7.37 (dd, *J* = 7.8 Hz and *J* = 1.6, 1H), 7.14–7.09 (m, 1H), 7.07–7.03 (m, 1H), 6.97 (d, *J* = 7.8, 1H), 6.50 (dd, *J* = 73.9 Hz and *J* = 76.7, 1H), 5.90 (br s, 1H), 5.31 (s, 1H), 4.69 and 4.63 (AB-system, *J* = 14.2, 2H), 4.01–3.91 (m, 2H), 3.54–3.49 (m, 2H), 3.26–3.21 (m, 2H), 2.34 (s, 3H), 2.30 (s, 3H), 1.66–1.58 (m, 2H), 1.57–1.49 (m, 6H), 1.30–1.20 (m, 18H), 0.88 (t, *J* = 6.8, 3H); ^13^C-NMR (CDCl_3_) 167.6, 167.1, 165.4, 149.5, 145.7, 144.2, 138.6, 131.9, 127.5, 125.0, 118.1, 117.06 (t, *J* = 254 Hz), 103.2, 101.8, 64.0, 61.9, 45.6, 43.0, 35.9, 31.9, 29.7, 29.6, 29.5, 29.4, 29.3, 29.2, 28.6, 26.2, 26.0, 25.3, 24.4, 22.7, 19.8, 19.4, 14.1; MS (+ESI) m/z (relative intensity) 633 ([M + H]^+^, 100). Anal. Calc. for C_35_H_50_F_2_N_2_O_6_: C 66.43; H 7.96; N 4.43; found: C 66.30; H 8.07; N 4.35.

### 3.4. General Procedure for the Aminolysis of Ethoxycarbonylmethyl 1,4-Dihydropyridine-3-Carboxylates ***3a,d*** when Amine is Used as Solvent/Nucleophile

1,4-DHP **3a**,**d** (1 equiv.) was dissolved in 15 fold excess of the appropriate amine at rt after which TBD (20 mol %) was added. The mixture was stirred at 30 °C for 3 or 4 h. The excess of amine was removed under reduced pressure and the residue was triturated with water. The white precipitate was filtered off and crystallised from diluted ethanol giving amides **4b**, **h**, **i**, **m**.

*3-Ethyl 5-(2-(isopropylamino)-2-oxoethyl) 2,6-dimethyl-4-phenyl-1,4-dihydropyridine-3,5-dicarboxylate* (**4b**). Yield 76%; light yellow powder; m.p. 115 °C; ^1^H- and ^13^C-NMR spectral data (CDCl_3_) were identical to that described above for **4b**.

*3-Ethyl 5-(2-oxo-2-(piperidin-1-yl)ethyl) 2,6-dimethyl-4-phenyl-1,4-dihydropyridine-3,5-dicarboxylate* (**4h**). Yield 85%; white crystals; m.p. 202 °C; ^1^H- and ^13^C-NMR spectral data (CDCl_3_) were identical to that described above for **4h**.

*3-Ethyl 5-(2-morpholino-2-oxoethyl) 2,6-dimethyl-4-phenyl-1,4-dihydropyridine-3,5-dicarboxylate* (**4i**). Yield 94%; white crystals; m.p. 185 °C; ^1^H- and ^13^C-NMR spectral data (CDCl_3_) were identical to that described above for **4i**.

*3-Dodecyl 5-(2-oxo-2-(piperidin-1-yl)ethyl) 4-(2-(difluoromethoxy)phenyl)-2,6-dimethyl-1,4-dihydro-pyridine-3,5-dicarboxylate* (**4m**). Yield 87%; light yellow powder; m.p. 96 °C; ^1^H- and ^13^C-NMR spectral data (CDCl_3_) were identical to that described above for **4m**.

### 3.5. 3-Carboxymethyl 5-Ethyl 2,6-dimethyl-4-phenyl-1,4-dihydropyridine 3,5-dicarboxylate *(**5a**)*

This compound was prepared via the method used for aminolysis of ethoxycarbonylmethyl esters of 1,4-dihydropyridines **3a**–**d** in DMF. Beginning with ester **3a** (1.16 g, 3 mmol), diisopropylamine (0.91 g, 1.26 mL, 9 mmol), TBD (84 mg, 20 mol %) and DMF (1 mL). The residue was dissolved in water (15 mL) and filtered off. The ice cooled filtrate was acidified with diluted hydrochloric acid to pH 4. The precipitated product was filtered, washed with cold water and crystallised from methanol yielding 0.75 g (70%) of compound **5a** as white crystals; mp 107–109 °C (dec). IR (film) 3346, 1744, 1680, 1640 cm^−1^. ^1^H-NMR (DMSO-*d*_6_) 12.90 (br s, 1H), 8.92 (s, 1H), 7.21–7.15 (m, 4H), 7.10–7.07 (m, 1H), 4.91 (s, 1H), 4.53 and 4.48 (AB-system, *J* = 15.7, 2H), 4.05–3.96 (m, 2H), 2.28 (s, 6H), 1.13 (t, *J =* 7.2, 3H); ^13^C-NMR (DMSO-*d*_6_) 170.0, 166.8, 166.3, 147.6, 146.6, 145.3, 127.8, 127.1, 125.9, 102.0, 100.7, 60.0, 59.2, 38.3, 18.4, 18.1, 14.2; MS (−ESI) *m*/*z* (relative intensity) 358 ([M − H]^−^, 80). Anal. Calc. for C_19_H_21_NO_6_: C, 63.50; H, 5.89; N, 3.90; found: C, 63.41; H, 5.85; N, 3.82.

## 4. Conclusions

The synthesis of carbamoylmethyl esters of dihydropyridines in direct aminolysis reaction of the appropriate ethoxycarbonylmethyl esters with primary and secondary alkylamines and cyclic amines under mild conditions in good to excellent yields in the presence of TBD was achieved. The usefulness of TBD as an efficient catalyst has been shown in the example of aminolysis reaction with secondary cyclic amines where from a number of *N*-containing bases tested, such as TEA, pyridine, DMAP, DBU, imidazole, TMG and MTBD, only TBD was found capable to catalyse this reaction. It should be admitted that aminolysis reactions of 1,4-DHP **3a**–**d** with secondary amines in the absence of TBD did not proceed at all and with primary ones aminolysis occurred with very low reaction rates. Aminolysis reactions of ethoxycarbonylmethyl ester of 1,4-dihydropyridines with 1-propyl-, 2-propyl- or 1-butylamine as well as secondary alkylamines and cyclic amines proceeded with good to excellent yields in all cases. It was also found that there is no significant influence of substituents at position 4 and the other ester moiety of 1,4-dihydropyridine on the aminolysis reaction with cyclic amines. The absence of aminolysis for ethoxycarbonylmethyl esters with sterically crowded secondary amines, such as diisopropylamine and diphenylamine or 1-naphtylamine was caused by steric hindrance of the bulky structure of the amines. Aminolysis reactions in all cases occurred regioselectively, as other alkyl esters of the 1,4-DHP molecule were not involved in the reaction. In the example of 2-propylamine, piperidine and morpholine it was shown that reactions can be performed both by using the reacting amine as solvent and nucleophile, however in this case a 15 fold excess of the amine should be used. In our view, it is more practical to perform aminolysis reactions in DMF as the amine in this case can be used in a reduced amount. The elaborated methodology of direct catalytic amide formation will certainly find its applications in the synthetic pathways for constructing of various heterocyclic compounds for further pharmacological studies.
